# Dimethylsulfoxide-Associated Anaphylaxis in Autologous Stem Cell Transplantation: A Case Report

**DOI:** 10.1155/carm/9351610

**Published:** 2025-10-24

**Authors:** Didar Yanardag Acik, Elif Suyani, Bilal Aygun

**Affiliations:** ^1^Department of Internal Medicine and Haematology, Faculty of Medicine, Health Sciences University Adana, Adana, Türkiye; ^2^Department of Internal Medicine and Haematology, Adana City Training and Research Hospital, Adana, Türkiye

**Keywords:** dimethylsulfoxide, intubation, life threatening anaphylaxis, management, stem cell transplantation

## Abstract

Dimethylsulfoxide (DMSO) is often used for freezing hematopoietic stem cells. DMSO is associated with mild side effects and rarely serious side effects such as anaphylaxis. We present a patient with acute anaphylaxis after DMSO and patient management.

## 1. Introduction

Dimethylsulfoxide (DMSO) prevents damage to hematopoietic stem cells during cryopreservation [1]. Autologous stem cell infusions cryopreserved with DMSO have been reported to cause a wide range of toxicities, mostly mild [2]. A life-threatening anaphylaxis-like reaction is very rare and has only been reported in isolated cases [3–7].

Anaphylaxis is a life-threatening medical condition by causing acute laryngeal edema. It may occur for many reasons including hypersensitivity to drugs, insect stings, and foods. Urgent medical intervention is required, and its treatment is rapidly administered epinephrine [8, 9].

In this article, we report a patient with DMSO-induced anaphylaxis during autologous stem cell infusion.

## 2. Cases

A 63-year-old man with chronic obstructive pulmonary disease (COPD) and diffuse large B-cell lymphoma (DLBCL) stage IV was treated with 6 cycles of rituximab, cyclophosphamide, adriamycin, vincristine, prednisone (R-CHOP) chemotherapy and 2 cycles of rituximab, etoposide, carboplatin, and ifosfamide (R-ICE) chemotherapy after relapse followed by autologous bone marrow transplantation for consolidation. Rituximab, carmustine, etoposide, cytarabine, melphalan (R-BEAM) preparation regimen was given. The patient had no previous history of allergy to drugs. 8 mg dexamethasone (iv) and 45.5 mg pheniramine hydrogen maleate (iv) were administered for premedication 30 min before stem cell infusion. Frozen stem cell infusion with DMSO was started, and 30–65 s after the infusion of 6–8 mL of product, the patient developed an allergic reaction, lost consciousness, and oxygen saturation dropped to 30%. In our center, patients who develop anaphylactic reactions are treated in accordance with the European Academy of Allergy, Asthma, and Clinical Immunology (EAACI) guidelines, and the same protocol was applied for this patient. The infusion was discontinued, adrenaline (0.5 mg, intramuscular) and prednol (80 mg, intravenous) were administered, and the patient was ambulated. With this intervention, the patient regained consciousness and oxygen saturation increased. The stem cells in the first bag were destroyed. However, since it was vital for the patient to receive stem cells, the remaining stem cells had to be infused. Because of the possibility of severe laryngeal edema in case of anaphylaxis developing again and intubation was not possible, the patient was put to sleep by the anesthesiologist in his room in the bone marrow transplant unit, and intubated and connected to a ventilator about 4 h later. 500 mg methylprednisolone (iv) was given, and stem cell infusion was restarted while the patient was connected to the ventilator. 4.7 × 10^6^/kg CD34 (+) stem cells were given, and vital signs were monitored. A urinary catheter was inserted, and urine output and urine color were monitored. The patient was intubated and ventilated for 48 h after stem cell infusion in case of possible reaction. Vital signs were stable. After 48 h, anesthetic drugs were discontinued, and the patient was awakened and extubated. The patient developed acute atrial fibrillation (AF) 5 days after transplantation, and cordarone and low molecular weight heparin were started by cardiology. AF resolved after pharmacologic cardioversion. The patient had neutrophil engraftment on Day +11 and platelet engraftment on Day +13 and was discharged on Day +18. A desensitization protocol was not implemented for our patient because there was no desensitization protocol for DMSO. The patient's adverse reaction, along with all procedures performed, was reported to the National Organ and Tissue Transplant Services Coordination Center (UKM) within the Ministry of Health.

### 2.1. Cell Therapy Product Information

The cell therapy product contained 8.7×10⁸ total nucleated cells (TNC) per kilogram and 4.7×10⁶ CD34 (+) cells per kilogram. The viability rate of CD34 (+) cells was 76%. The total product volume was 610 mL, including 30 mL of 5% DMSO. The additional components of the product were 78% patient plasma, 12% hydroxyethyl starch (HES), and 10% DMSO.

The patient's body weight is 82 kg. A total of 0.048 mL/kg DMSO was given in the first product applied to the patient. The DMSO (lot no: W21553) was obtained from Origen Biomedical, which held a Conformité Européenne (CE) certificate. It has International Organization for Standardization (ISO) 13,485; 2016 and Medical Device Single Audit Program (MDSAP) certificates. It is stored in the dark at 20–30 0 C. There is no need for a cold chain. The DMSO supplier was notified. It was learned that there were no adverse reaction reports with the same batch of DMSO. No adverse reactions were observed in our other patients who underwent transplantation with the same lot number.

## 3. Discussion

DMSO is the cryopreservation agent most frequently used by transplant centers for stem cell freezing and storage [9]. Non-life-threatening side effects are seen with DMSO-containing transplants. The most common are nausea, vomiting, hypertension/hypotension, sedation, or headache [10]. Although low doses do not cause many side effects, significant neurological, cardiac, and respiratory side effects have been reported in the literature [9, 11–13]. Neurological side effects are due to DMSO crossing the blood–brain barrier [14]. Some authors have suggested washing away DMSO before infusion to reduce side effects [9], while others have argued that washing may cause stem cell loss and decreased viability [10].

Morris et al. prospectively studied side effects in 64 patients who underwent autologous stem cell transplantation with DMSO-frozen stem cells at 64 European Blood and Marrow Transplant Group centers. They reported that most of these centers used DMSO at a concentration of 10%, some used DMSO at 7.5% and 5% concentrations, and that fewer side effects were observed with lower DMSO concentrations. In addition, some of these centers have implemented a stem cell washing protocol before infusion to reduce side effects [15]. Khan et al. stated that they could not find any relationship between the amount of DMSO administered and side effects [16].

In the case reported by Benekli et al., immediately after infusion of DMSO-frozen stem cells, the patient developed acute restlessness, fixed gaze, and then respiratory depression, leading to complete arrest without spontaneous breathing. The infusion was stopped immediately, and vital signs stabilized without the need for intubation. DMSO was then washed out, and stem cell infusion was performed, and the patient experienced no reaction [3].

In allogeneic transplantation, stem cells are administered simultaneously without freezing. However, umbilical cord-derived stem cells and allogeneic stem cells are frozen in cases where simultaneous transplantation is not possible. Koken et al. reported two pediatric cases of anaphylaxis following allogeneic transplantation. In these transplants, the stem cells were frozen, but they did not specify whether the anaphylaxis was due to DMSO or another component of the stem cells. However, in one case, the infusion was administered without complications after removing the DMSO from the remaining products [17].

Cases of anaphylaxis have also been reported in transplants performed without DMSO administration. Alloimmunization has been implicated in anaphylaxis independent of DMSO. In these cases, desensitization along with premedication is recommended [18, 19].

In our case, rinsing the DMSO before administering it to the patient could have been an option. However, this was not implemented because experienced personnel were unavailable, and it could have caused stem cell loss. Because the rooms in our bone marrow transplant unit have the necessary infrastructure to operate mechanical ventilators, we chose to monitor the patient with a mechanical ventilator without removing him from the bone marrow transplant unit. However, these facilities may not be available in all transplant units. Washing away DMSO before infusion is another option.

In conclusion, we can recommend that clinicians working in transplant clinics intubate the patient and give stem cell infusion as a safer method for a patient who develops anaphylaxis.
